# Seismic performance analysis of shallow-buried large-scale three-box-section segment-connected pipeline structure under multiple actions

**DOI:** 10.1038/s41598-023-28516-0

**Published:** 2023-02-14

**Authors:** Hui Wang, Zhen Chen, Shujiang Ma, Xiaoke Li, Shiming Liu, Shunbo Zhao

**Affiliations:** 1 School of Civil Engineering and Communications, North China University of Water Resources and Electric Power, Zhengzhou, 450045 China; 2 International Joint Research Lab for Eco-Building Materials and Engineering of Henan, North China University of Water Resources and Electric Power, Zhengzhou, 450045 China; 3 Hebei Water Resources Planning, Design and Research Co. LTD, Shijiazhuang, 450046 China; 4 Collaborative Innovation Center for Efficient Utilization of Water Resources, North China University of Water Resources and Electric Power, Zhengzhou, 450046 China

**Keywords:** Engineering, Mathematics and computing

## Abstract

To ensure the safety operation of inter-basin water transfer project, it is important to understand the seismic performance of shallow-buried large-scale segment-connected pipeline structure under earthquake and other multiple actions. In this paper, a 3D FE model is built in details for the seismic assessment of large-scale prestressed concrete inverted-siphon shallow-buried under riverbed, and a method is proposed to realize multiple interactions of the viscoelastic boundary elements to finite elements, the compressible fluid to structure and the sealed joints made of rubber belt between adjacent segments. The inverted-siphon is longitudinally composited by 58 segments in total length of 858.64 m, and transversally consisits of three rectanguler-section boxes with net dimension of each box of 6.5 m width and 6.6 m height. The main influencing factors are set as the thickness of overburden soil, the flow condition and the engineering geological condition. The time-history analysis is carried out on the 3D FE model under Elcentro seismic wave excitation. The numerical analyses figures out the main vibration modes and vibrational frequency of this inverted-siphon, reveals the regions of peak tensile stress appeared on the concrete surface and the risk sealed joints with extremum relative displacement. This provides a scientific reference to take specific prevention measures of anti-earthquake, based on a deep understanding to the seismic cracking of concrete and the tearing broken of sealed joints for this kind of segment-connected pipeline structure.

## Introduction

Water is the source of live, production and ecosystem. To solve serious imbalance between supply and demand of water for different population areas, large-scale and long-distance water transfer projects have been built over China, including the Water Supply Project from Dongjiang River to Shenzhen and Hong Kong^1^, the Water Supply Project from Dahuofang Reservoir to Shenyang and Dalian^2^, and the South-to-North Water Transfer Project from Danjiangkou Reservoir to Beijing^3^. In these projects, the segment-connected pipeline structures have been commonly applied. Among them, the inverted-siphon is one of the main structures across the rivers and valleys. During the design for inverted-siphon engineering structure, except for concerning about the static performance^3–6^, more attention have been payed on the flow dynamics^6–8^, the operating safety due to ice jam in period of winter^9–11^. Where only one or several segments of the inverted-siphon are considered for investigation. On the basis of China code NB 35047^12^, the special research should be carried out by using the spatial structure analytical model to calculate the seismic response of underground hydraulic structure with complex geological conditions, where the dynamic interaction between the structure and the surrounding soil should be included. This reminds that the seismic behaviors of shallow-buried large-scale inverted-siphon is an issue needed to be researched to the safty operation of water transfer project.

However, the research faces a very complicated matter to reveal the seismic performance of large-scale inverted-siphon composited by connected-segments with sealed-joints of rubber belt which is long-distance shallow-buried on different soil foundation.

Firstly, due to the experimental study is difficult to be achieved, this needs to bulid a comprehensive numerical model of large-scale inverted-siphon with tremendous volume composited by multiple connected-segements which are long-distance shallow-buried with different surrounded soils. The best way is by using Finite Element (FE) method^13,14^. To minimize the computation work, the semi-infinite model of foundation should be transferred to the FE model with artificial boundary, and the earthquake input should be simulated on the artificial boundaries. Now, some artificial boundaries can be coupled with FE such as the viscous boundary^15,16^, the visco-elastic boundary^17^ and the artificial transmission boundary^18^. The visco-elastic boundary overcomes the low frequency drifting of viscous boundary and can simulate the elastic recovery capacity of semi-infinite medium outside the artificial boundary^19^, which is applied by many general FE softwares. Du and Zhao^20^ applied the visco-elastic boundary with FE model to analyze the seismic response of arch dam-foundation system, and made the earthquake input of foundation boundaries by the loading mode on boundary nodes. Ma et al.^21^ proposed the setting method of visco-elastic boundary for deep underground cavern group. He et al.^22,23^ presented the three-dimensional analytical method to predict ground vibrations induced by underground railways from a tunnel in a multi-layered half-space and two parallel tunnels embedded in a full-space. The tunnel is coupled with the multi-layered soil and the boundary conditions on the tunnel-soil interfaces are satisfied by the transformation between the cylindrical waves and plane waves.

Secondly, to reflect the seismic response of inverted-siphon in water delivery condition, the interaction between water and structure should be considered. The method by using the additional mass to simulate the hydrodynamic pressure, proposed by Wsetergaard^24^, is widely utilized, however the compressibility and shaking effect of water could not be reflected^25^. Chopra^26^ discovered that compared to the additional mass in FE models, obvious differential of hydrodynamic pressure existed if the compressibility of water was considered. Li and Gong^27,28^ respectively adopted the displacement-displacement format and the displacement–pressure format to realize the earthquake analysis for the hydraulic structure-compressible fluid interaction models. Elansary et al.^29^ conducted the seismic analysis of liquid storage composite conical tanks by using an in-house developed model that accounts for the hydrodynamic pressure resulting from the vibration of the contained fluid, and the interaction between the fluid and the structure vibrations. These achievements have been successfully applied for the analyese of the seismic behaviors of concrete dams^30–32^, and other hydraulic concrete structures^33,34^.

Meanwhile, Ding et al.^35^ proposed a model for predicting dynamic response of composite lining in saturated poroelastic medium under incident P wave based on nonlocal-Biot theory, and provided the analytical solution by using wave function expansion method. The size effect described by the nonlocal parameter on dynamic stress concentration of composite lining is studied in detail, and the influences of input frequency, incident angle, thickness of composite lining, stiffness of composite lining and the buried depth of the lining on dynamic stress concentration are elaborated to obtain the distribution pattern of dynamic stress concentration factor for safety design in tunnel engineering.

Motivated by no detailed research being done and the lack of information about seismic behavior of shallow-buried large-scale prestressed concrete inverted-siphon composited by connected-segments with sealed-joints of rubber belt, an comprehensively analytical research is conducted by using the FE method with the combination of the technologies of boundary interaction, fluid–structure interaction and joints interaction. The boundary characteristics, the water delivery function and the feature of multi-segments linked by sealed joints are simulated in detail. The peak-stress of inverted-siphon concrete and the extremum relative displacement between adjacent segments are inverstigated as the key points, and the possibilities of concrete cracking and water leakage due to earthquake damage are evaluated. This is of great value to deeply understand the seismic performance and take specific prevention measures for this kind of segment-connected pipeline structures.

## FE method for seismic analysis of inverted-siphon engineering

### Description of inverted-siphon structure

**Figure 1:**
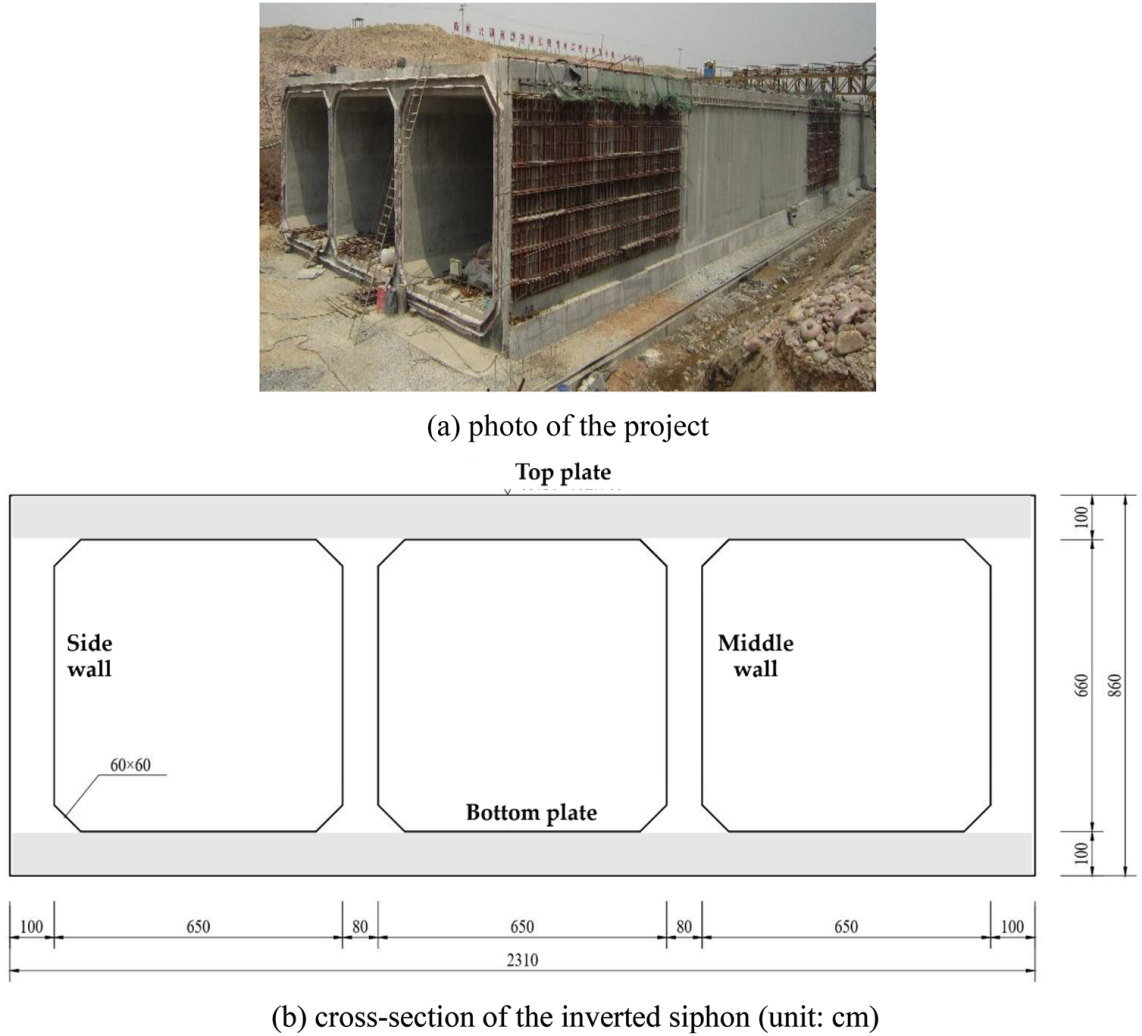
A project of inverted-siphon in construction. (**a**) photo of the project. (**b**) Cross-section of the inverted siphon (unit: cm).

An prestressed concrete inverted-siphon was designed for the Central Route of South-to-North Water Transfer Project, China, which consists of three boxes with transversal rectangular-section and is longitudinally composited by 58 segments connected by the sealed joints made of rubber belt^3^. As shown in Fig. 1, it is 858.64 m long in total including the inclined segments of entrance and exit and the flat ones under river course. The whole section with three boxes is 8.6 m height and 23.1 m wide, the flow section of each box is 6.5 m wide and 6.6 m height,. The thickness of bottom-plate, top-plate and side wall is 1.0 m respectively, and that of middle wall is 0.8 m.

The strength grade of concrete is C40 with the unit weight of 24.5kN/m^3^, the dynamic modulus of elasticity of 42.6 GPa, the Poisson’s ratio of 0.167, the axial compressive strength of 26.8 MPa and the tensile strength of 2.39 MPa^36^.

The engineering geological condition varies in multiple layers as loam, sandy loam, gravel, clay, sand with different fineness. The main properties of the soils are summarized in Table 1. The foundation ditch of the inverted-siphon was excavated firstly and backfilled by sandy gravel. At the ends of entrance and exit, the inclined segments were capped by the river dikes.

**Table 1:** Main physical and mechanical properties of soils.

Soil type	Mass density (kg/m^3^)	Poisson's ratio	Cohesion (kPa)	Frictional angle (°)	Elastic modulus (MPa)
Loam	1980	0.385	35.9	22.0	9.1
Sandy loam	1960	0.360	6.1	23.2	8.9
Gravel	2600	0.299	0.0	35.0	55.0
Clay	2040	0.350	54.2	14.5	14.6
Medium sand	1990	0.404	15.9	24.2	6.2
Silty sand	1980	0.250	16.2	19.2	6.0
Coarse sand	1990	0.404	15.9	24.2	6.2
Backfill sandy gravel	2200	0.350	10.0	35.0	23.0

### FE model of inverted-siphon engineering

The FE model of inverted-siphon is built to simulate the real structure and engineering geological condition. Considered the impact of surrounding soils with the size that is usually 3 to 5 times the size of inverted-siphon structure in the same direction, the width of surrounding soils is set as 160 m while the thickness under the bottom-plate is set as 30 m. The structure of inverted-siphon and the surrounding soil are simulated by Solid45 elements, and the water flow is simulated by Fluid30 elements. The elastoplasticity of surrounding soil is determined by Drucker-Prager yield criterion. The contact surface of fluid–structure interaction is assigned by FSI label. The ground surface is free, and other directions of surrounding soils are simulated as viscoelastic boundary by the spring-damper element Combin14 with APDL program.

**Figure 2:**
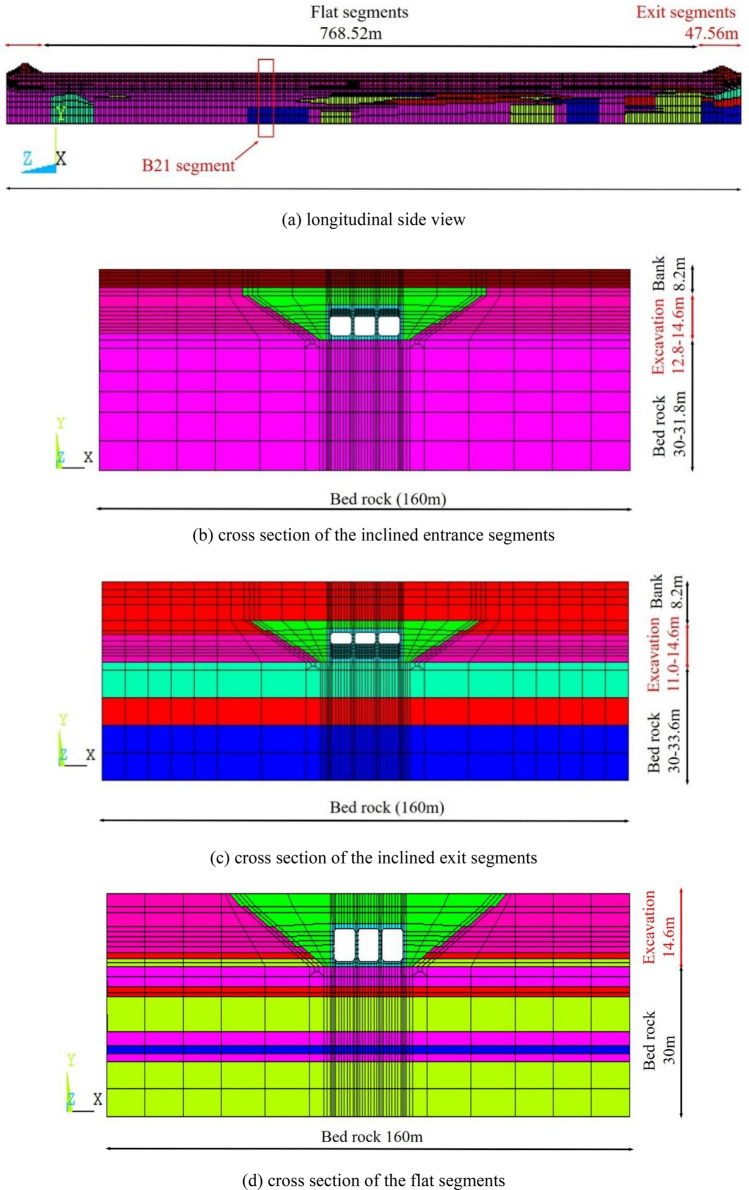
FEM for inverted siphon and bedsoil. (**a**) Longitudinal side view. (**b**) Cross section of the inclined entrance segments. (**c**) Cross section of the inclined exit segments. (**d**) Cross section of the flat segments.

The FE model of the inverted-siphon includes about 200 thousand elements and 250 thousand nodes. As the whole inverted-siphon crosses a long distance of 858.64 m, the B21 segment on choppy basement is selected as the main segment to be studied and its element is refined. Figure 2 exhibits the longitudinal side view of the entire model, the cross section of inclined entrance segments, the cross section of inclined exit segments and the cross section of the flat segments under river course. The coordinate system is built with X*-*axis along the width, Y*-*axis along the height and Z*-*axis along the length.

The analyses include the dynamic characteristics and seismic behaviors of inverted-siphon structure. According to China code SL191^36^ and combined with the influence factors of channel water and overburden backfilling, three operating conditions are considered as follow:

Case I: no water in river course, water flow in three boxes and 0.5 m of minimum overburden soil;

Case II: no water in river course, water flow in middle box and 6 m of maximum overburden soil;

Case III: no water in river course, water flow in two side boxes and 6 m of maximum overburden soil.

**Figure 3:**
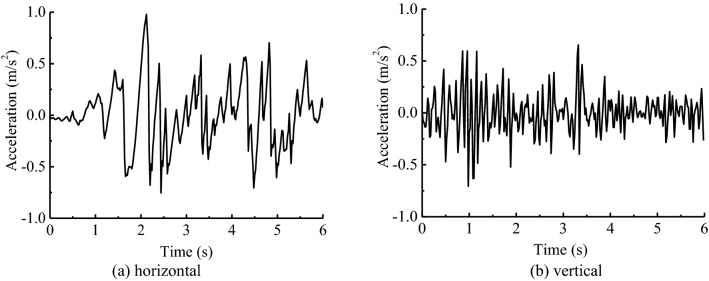
Acceleration time-history curves of Elcentro seismic wave. (**a**) Horizontal (**b**) Vertical.

After the balance of initial geostress considering the self-weight of inverted-siphon structure and surrounding soil, and the hydrostatic pressure in the box, the time-history analyses are made under the seismic excitation along X*-*axis and Y*-*axis. The seismic fortification intensity is chosen as VII degree, the horizontal and vertical seismic peak acceleration is 0.10 g and 0.067 g, respectively. Seismic waves modulated by Elcentro seismic waves are presented in Fig. 3. The total calculation time is 6 s with steps of 0.02 s. The time-histories of velocity and displacement are given from the integration of acceleration time-history, and the wave excitation is realized by the input of equivalent seismic loads on nodes of the bottom boundary^37,38^.

### Principle of interaction between viscoelastic BE and FE

**Figure 4:**
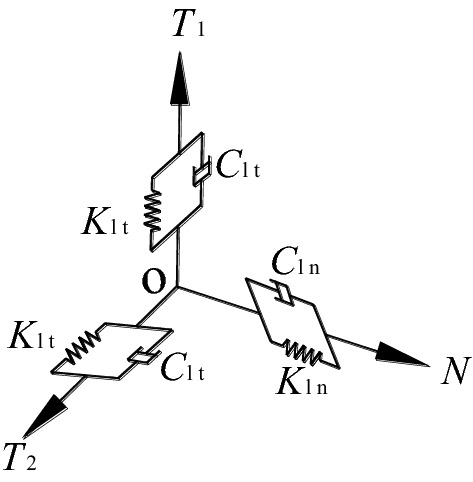
3D spring-damper system of artificial boundary node.

3D viscoelastic Boundary Element (BE) is set parallel connected spring-damper system on the FE artificial boundary, as presented in Fig. 4. It leads the same stress state on boundary to theoretical solution of wave equation under the same wave, and realizes the simulation of infinite wave field. Where, *T*_1_ and *T*_2_ respectively represent the tangent directions of the model boundary surface, and *N* represents the normal direction.

The spring stiffness and damper coefficient of artificial node along normal and tangent directions are calculated as follows^39–41^:

1 $$K_{{{\text{ln}}}} = \alpha_{{\text{n}}} \cdot \frac{G}{R}$$

2 $$C_{{{\text{ln}}}} = \rho \cdot c_{{\text{p}}}$$

3 $$K_{{{\text{lt}}}} = \alpha_{{\text{t}}} \cdot \frac{G}{R}$$

4 $$C_{{{\text{lt}}}} = \rho \cdot c_{{\text{s}}}$$

where *K*_1n_ and *K*_1t_ are the spring stiffness along normal and tangent directions, respectively;* C*_1n_ and *C*_1t_ are the damper coefficient along normal and tangent directions, respectively; *G* and *ρ* are shear modulus and mass density of media; *R* is the distance of wave source to artificial boundary; *c*_p_ and *c*_s_ are the wave velocity of media’ P-wave and S-wave; *α*_n_ and *α*_t_ are correction coefficients of viscoelastic artificial boundary along normal and tangent directions which are taken as 4 and 2 respectively for 3D FE model.

Taken only travelling wave close to the field into account, the FE equations of motion at *l* node in *i* direction are presented as follows^41^:

5 $$m_{{\text{l}}} \ddot{u}_{{{\text{li}}}} + \sum\limits_{k = 1}^{n} {\sum\limits_{j = 1}^{n} {(c_{{{\text{likj}}}} + \delta_{{{\text{lk}}}} \delta_{{{\text{ij}}}} A_{{\text{l}}} C_{{{\text{li}}}} )\dot{u}_{{{\text{kj}}}} } } + \sum\limits_{k = 1}^{{n_{{}} }} {\sum\limits_{j = 1}^{n} {(k_{{{\text{likj}}}} + \delta_{{{\text{lk}}}} \delta_{{{\text{ij}}}} A_{{\text{l}}} K_{{{\text{li}}}} )\mathop u_{{{\text{kj}}}} } } = f_{{{\text{li}}}}$$

6 $$f_{{{\text{li}}}} = A_{{\text{l}}} \left( {K_{{{\text{li}}}} u_{{{\text{li}}}}^{{\text{R}}} + C_{{{\text{li}}}} \dot{u}_{{{\text{li}}}}^{{\text{R}}} + \sigma_{{{\text{li}}}}^{{\text{R}}} } \right)$$

where *m*_l_ is the concentrated mass of *l* node; *ü*_li_ is the acceleration of *l* node along the* i* direction; ù_kj_ and* u*_kj_ are the velocity and displacement of *k* node along the *j* direction; *δ*_ij_ = 1 (i = j), *δ*_ij_ = 0 (i ≠ j); *C*_likj_ and *K*_likj_ are the damping and spring stiffness coefficients of *k* node along the *j* direction relative to *l* node along the *i* direction, respectively; *C*_li_ and *K*_li_ are the damping and spring stiffness coefficients of *l* node along the *i* direction at the artificial boundary; *A*_l_ is the influenced area of *l* node; *f*_li_ is the equivalent node force of *l* node along *i* direction under incident wave, i.e. the resistance required to overcome the spring, damper and media.

Suppose the displacement time-history of vertical incident P-wave and S-wave on the bottom boundary are *u*_lp_(t) and *u*_ls_(t), respectively, the equivalent node force of *l* node calculated from formula (6) are:

7 $$f_{{{\text{ly}}}}^{{\text{ - y}}} \left( t \right) = A_{{\text{l}}} \left[ {K_{\ln } u_{{{\text{lp}}}} (t) + C_{{{\text{ln}}}}^{{}} \dot{u}_{{{\text{lp}}}} (t) + \rho c_{{\text{p}}} \dot{u}_{{{\text{lp}}}} (t)} \right]\quad \left( {{\text{incident P}} - {\text{wave}}} \right)$$

8 $$f_{{{\text{lx}}}}^{{\text{ - y}}} \left( t \right) = A_{{\text{l}}} \left[ {K_{{{\text{lt}}}} u_{{{\text{ls}}}} (t) + C_{{{\text{lt}}}}^{{}} \dot{u}_{{{\text{ls}}}} (t) + \rho c_{{\text{s}}} \dot{u}_{{{\text{ls}}}} (t)} \right]\quad \left( {{\text{incident S}} - {\text{wave}}} \right)$$

where the subscripts of equivalent node force represent the node number and the force direction, the superscript represents the outward normal direction of artificial boundary of the node located.

### Principle of water-structure interaction

Based on the displacement–pressure (*u*, *p*) format of Euler-method, the water-structure interaction is described with the displacement *u* for the inverted-siphon and the pressure *p* for the water flow^30,31^. Taken the advantage of the similarity of motion equations of water and elastic structure, the computation mode of water FE can be obtained corresponded to that of structural FE. As only one degree of freedom of the pressure on water node, the relatively reduced degree of freedom in the water body leads a higher computing efficiency compared to the hydrodynamics formulation with three displacements. The body of water is supposed as compressible, non-viscosity and homogeneous.

For the water element, the equation with *p* as the target can be expressed as:

9 $$\frac{1}{{c^{2} }}\ddot{p} - \nabla p = 0$$

where $c = \sqrt {{k \mathord{\left/ {\vphantom {k \rho }} \right. \kern-0pt} \rho }}$ is the sound velocity in the body of water, *k* is the volume modulus of the body of water, *ρ* is the density of water, *p* is the hydrodynamic pressures.

The boundary condition on the interface between inverted-siphon and water is:

10 $$\frac{\partial p}{{\partial n}} = - \rho \dot{u}_{{{\text{in}}}}$$

where* p* is the hydrodynamic pressures on node of interface, $\dot{u}_{{{\text{in}}}}$ is the normal component of water body acceleration.

Therefore, the dynamic equilibrium equation can be presented with the block matrix as:

11 $$\left[ {\begin{array}{*{20}c} M & 0 \\ {\rho Q} & {M^{\prime}} \\ \end{array} } \right]\left\{ {\begin{array}{*{20}c} {\ddot{u}} \\ {\ddot{p}} \\ \end{array} } \right\} + \left[ {\begin{array}{*{20}c} C & 0 \\ 0 & {C^{\prime}} \\ \end{array} } \right]\left\{ {\begin{array}{*{20}c} {\dot{u}} \\ {\dot{p}} \\ \end{array} } \right\} + \left[ {\begin{array}{*{20}c} K & { - Q} \\ 0 & {K^{\prime}} \\ \end{array} } \right]\left\{ {\begin{array}{*{20}c} u \\ p \\ \end{array} } \right\} = \left\{ {\begin{array}{*{20}c} f \\ {f^{\prime}} \\ \end{array} } \right\}$$

where *M*, *C*, *K* and *M*′, *C*′, *K*′ are the mass matrix, damper matrix and stiffness matrix of the inverted-siphon and the body of water, respectively; *Q* is the interaction matrix of the water-structure interface; *u* and *p* are the vectors of displacements and hydrodynamic pressures; $\dot{u}$, $\dot{p}$ and $\ddot{u}$,$\ddot{p}$ are the first and second derivatives of displacement and hydrodynamic pressure, respectively with respect to time; *f* is the sum of the all external forces and ground acceleration on structure; $f^{\prime}$ is the sum of the forces due to ground shaking on the solid–fluid boundaries and the total acceleration on the rest of the boundaries.

### Principle of segment interaction

Between the adjacent segments of inverted-siphon structure, the sealed-joint is constructed by the rubber belt of 20 mm thick. If the very thin layers of sealed joints and surrounding soil are modified, the high deformity elements would appear with terrific width-to-thickness ratio. Therefore, the thinner layer of surrounding soil is ignored, the segment interaction among nodes of surrounding soil is realized by the mode of constraint equation’ interaction.

## Analytical results and discussion

### Dynamic characteristics of inverted-siphon

**Figure 5:**
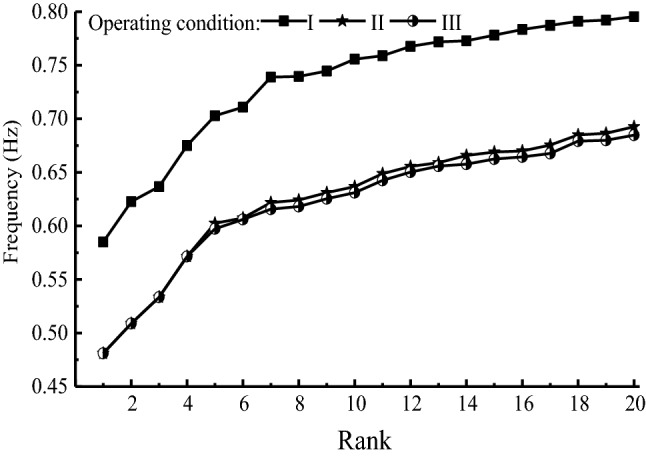
Vibrational frequency of the former 20 ranks.

Figure 5 presents the vibrational frequency curves of former 20 ranks. The frequency increases with the rank for every operating conditions. In case I, although the water flows in all boxes of inverted-siphon, the frequency of each rank is higher due to the minimum overburden soil. Due to the small water flow, the frequency of each rank in case II is slightly higher than that in case III. The vibration of inverted-siphon is obviously affected by the thickness of overburden soil, the vibrational frequency increases with the decreased thickness of overburden soil. The water flow also clearly affects on the vibration of inverted-siphon, but the vibrational frequency increases slightly with the decrease of water flow.

**Figure 6:**
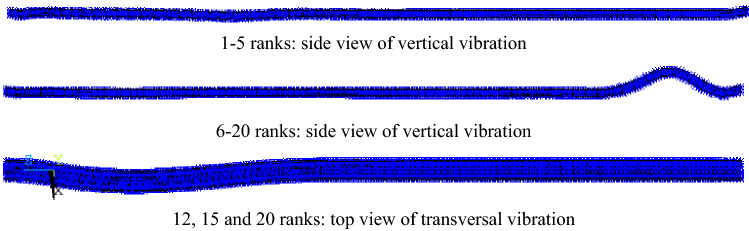
Main structural vibration modes of the former 20 ranks at case I (Enlarging proportion: 300 thousand for 1–5 ranks, 150 thousand for 6–20 ranks). 1–5 ranks: side view of vertical vibration. 6–20 ranks: side view of vertical vibration. 12, 15 and 20 ranks: top view of transversal vibration.

Under the three operating conditions, vertical vibration takes place in 17th rank, and transversal vibration takes place in other 3 ranks of the former 20 ranks. In case I, the whole vertical vibration first takes place, then the local vertical vibration, and finally the whole transverse vibration, as presented in Fig. 6. As the larger rigidity of inverted siphon than surrounding soil, the vibration modes present the relative deformation between segments rather than the local deformation of the plates. Due to the increased thickness of overburden soil in case II and case III, the vibration amplitudes decrease accompanied with a delayed transversal vibration. However, both of the vibration modes are quite similar only with slight differences of vibration amplitude. This indicates the small effect of water flow on the vibration mode.

### Seismic stress of concrete

**Figure 7:**
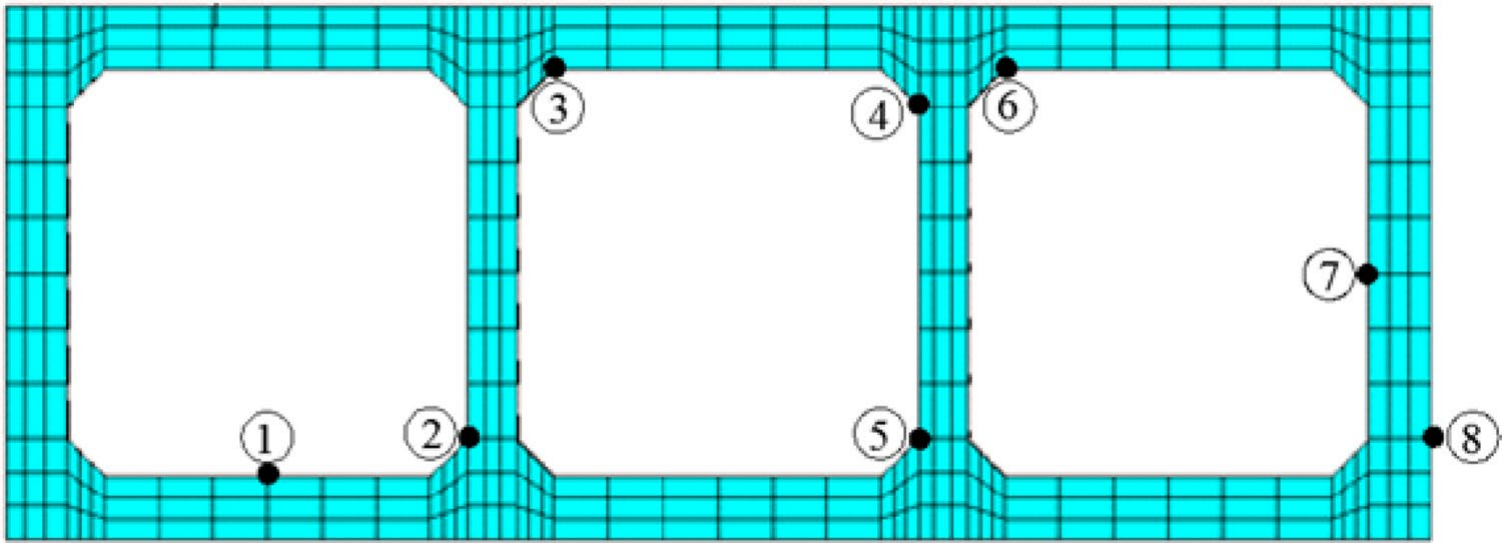
Cross-section and key nodes of inverted-siphon.

Taking B21 segment as the research object, the peak-stresses at key nodes of its middle cross-section in the whole time-domain are compared under three operating conditions. Table 2 presents the results with the compressive stress in negative and the tensile stress in positive. Figure 7 gives the location of the key nodes. For the walls of inverted-siphon, the compressive peak-stresses of nodes are 3.33 MPa and 3.52 MPa, respectively under case II and case III, and obviously higher than that under case I; the tensile peak-stresses are close to 0.8 MPa under the three cases. For the top and bottom plates, the tensile peak-stress appears on node ① at upper-surface of bottom-plate, while the compressive peak-stress appears on nodes ③ and ⑥ at bottom-surface of top-plate. The compressive stresses under case II and case III are 1 ~ 2 times higher than that of case I, the peak is 3.55 MPa. The tensile stresses under case II and case III are 1 time higher than that of case I, the peak is 3.58 MPa.

**Table 2:** Peak-stress σ_x_ and σ_y_ at key nodes in the whole time-domain.

Structural member		Case I		Case II		Case III	
		Key node	Stress (MPa)	Key node	Stress (MPa)	Key node	Stress (MPa)
Wall	σ_y,min_	⑦	− 1.99	②	− 3.33	⑤	− 3.52
	σ_y,max_	④	0.80	⑧	0.80	⑧	0.75
Plate	σ_x,min_	③	− 1.18	⑥	− 2.74	③	− 3.55
	σ_x,max_	①	1.72	①	3.58	①	3.07

**Figure 8:**
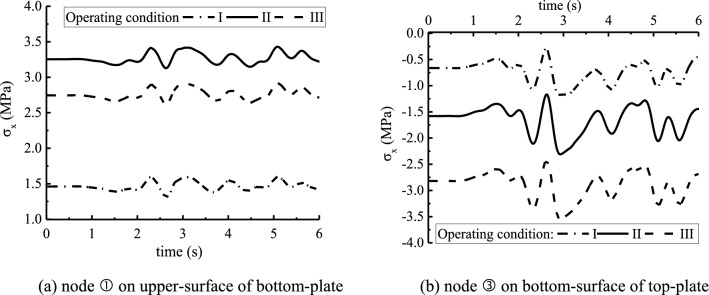
Stress time-history curves of key nodes on plates. (**a**) Node ① on upper-surface of bottom-plate (**b**) node ③ on bottom-surface of top-plate.

Figure 8 draws the stress time-history curves for the controlled key nodes ① and ③ on the plates. The variations of stresses are close to each other under the three cases, and the fluctuations appear obviously at 2.6 s and reach the peak value. This is slightly delayed compared to the peak of seismic wave. For case I, the average tensile stress of node ① is 1.5 MPa while the average compressive stress of node ③ is 0.7 MPa, the stresses keep at a lower level. For cases II and III, the average tensile stress of node ① is about 3.3 MPa and 2.7 MPa respectively, while the average compressive stress of node ③ is about 1.6 MPa and 2.8 MPa. Comparatively, the water flow in three boxes at case I benefits to the resisting of seismic action. If the earthquake takes place during the period of maintenance, the water flow in two side boxes at case III is better than that in middle box at case II.

**Figure 9:**
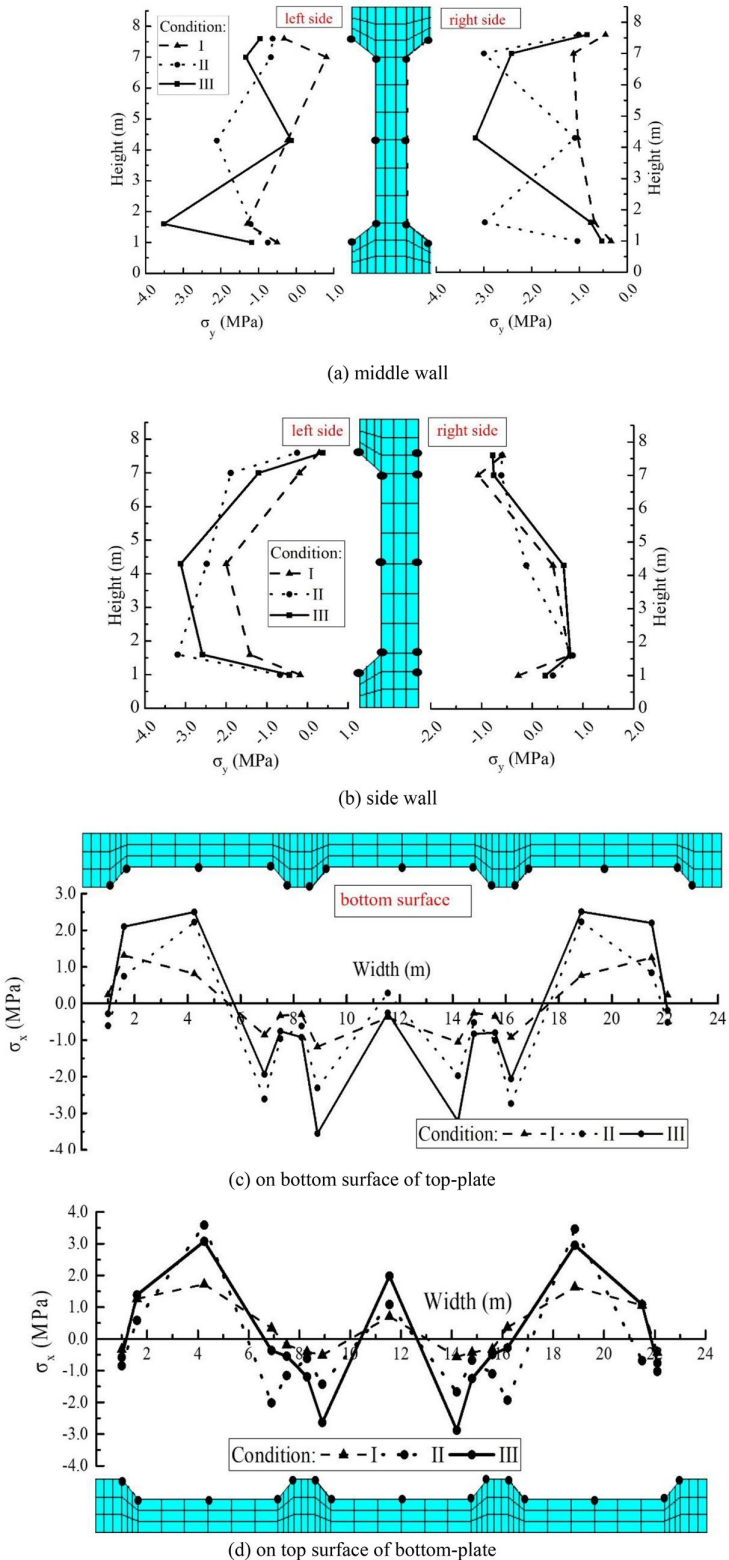
Extremum stress distribution on main surfaces of inverted-siphon. (**a**) middle wall, (**b**) side wall, (**c**) on bottom surface of top-plate, (**d**) on top surface of bottom-plate.

Figure 9 exhibits the peak-stress distribution on main surfaces of inverted-siphon structure. Except the small tensile stress appeared on the outside surface of side walls, the walls are all in compression. In the corner from wall to plate, a larger change of stress exists due to the stress concentration. The peak-tensile stress is about 0.8 MPa. This displays that the earthquake does not lead a higher tensile stress in walls of inverted-siphon. Under the three cases, large tensile stresses appear on the bottom-surface of top-plate in side boxes and the upper-surface of bottom-plate in three boxes. This may result in the concrete cracks due to the tensile stress over the tensile strength of C40 concrete.

### Seismic displacement of sealed joints

The sealed-joint between segments is the rubber belt in thickness of 20 mm. Under the seismic action, if the relative displacement of adjacent segments is so large that the tearing of rubber belt occurs, the sealed-joint will lose efficacy and affect the normal operating of water delivery. Therefore, it is necessary to track the relative displacement of adjacent segments. The four corner points of each segment end-section, such as the top left and right corner points, and the bottom left and right corner points, are taken as the key points. The relative displacements of these points between adjacent segments are compared in vertical and transversal directions, and the curves can be drawn to reflect the changes of relative displacement. The positive represents the relative displacement in upward vertical direction and that in leftward transversal direction.

**Figure 10:**
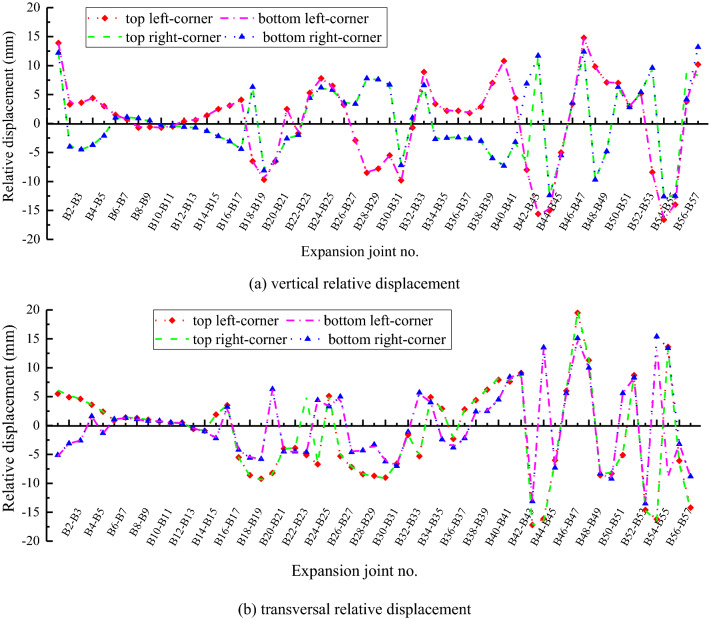
Relative displacement between segments of inverted-siphon at case I. (**a**) vertical relative displacement, (**b**) transversal relative displacement.

As presented in Fig. 10, the extremum vertical relative displacement is 13.9 mm between B1 inclined segment and B2 flat segment at case I, while it is within 5 mm before the B20 flat segments. The relative displacement obviously increases due to the complex changes of foundation layers after B43 segment. It is 14.8 mm between B47 and B48 segments, and −16.6 mm between B54 and B55 segments. Similarly, the extremum transversal relative displacement is within 5 mm before the B20 segments, while it is −17.5 mm between B43 and B44 segments, and 19.8 mm between B47 and B48 segments. The extremum relative displacement is −14.6 mm between the flat and inclined segments at the exit.

**Figure 11:**
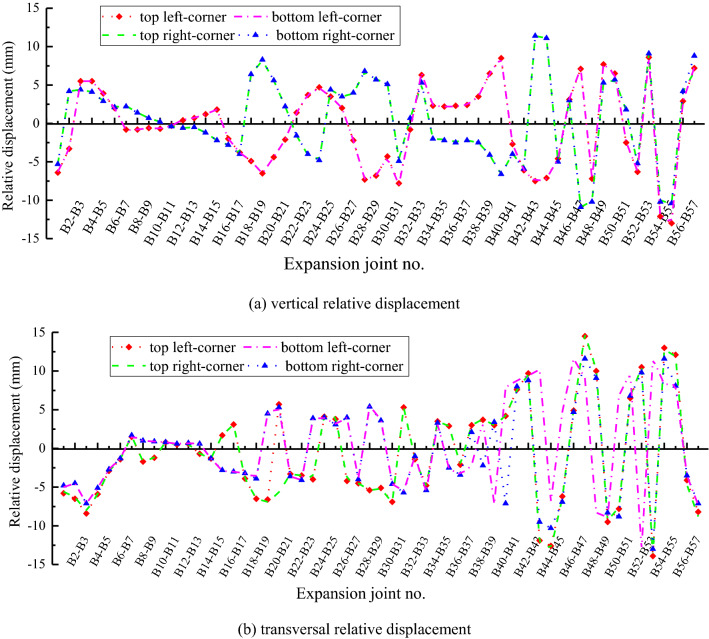
Relative displacement between segments of inverted-siphon at case II. (**a**) vertical relative displacement (**b**) transversal relative displacement.

As presented in Fig. 11, the extremum vertical relative displacement is within 4 mm before the B20 flat segments, and 11.4 mm between B43 and B44 segments at case II. It reaches −13.0 mm between segments close to river bank, and 8.8 mm between the flat and inclined segments at the exit. Similarly, the extremum transversal relative displacement is within 3.9 mm before the B20 segments, while it is -14.8 mm between B47 and B48 segments, and 13.9 mm between B54 and B55 segments. The extremum relative displacement is −8.8 mm between the flat and inclined segments at the exit.

**Figure 12:**
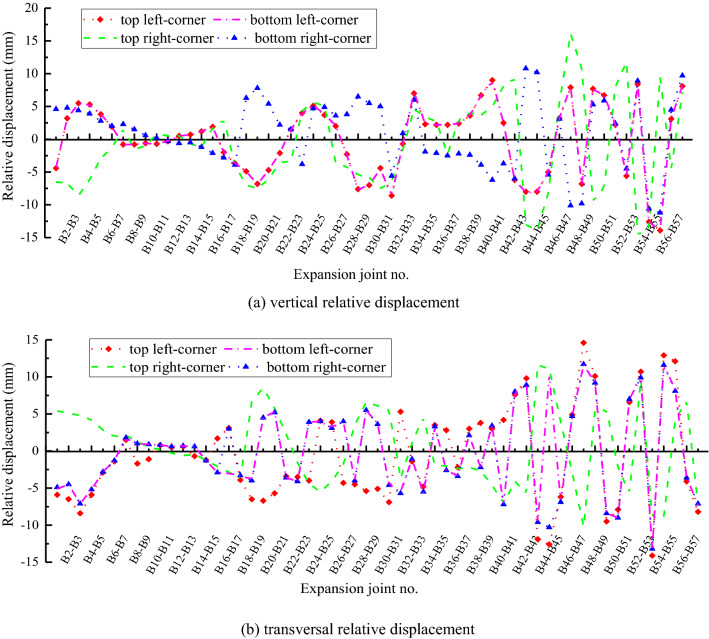
Relative displacement between segments of inverted-siphon at case III. (**a**) vertical relative displacement (**b**) transversal relative displacement.

As presented in Fig. 12, the extremum vertical relative displacement is within 3.8 mm before the B20 flat segments at case III. It is 16.1 mm between B47 and B48 segments, −14.7 mm between B53 and B54 segments, and 9.7 mm between the flat and inclined segments at the exit. Similarly, the extremum transversal relative displacement is within 3.0 mm before the B20 segments, while it is 14.6 mm between B47 and B48 segments, and 14.1 mm between B54 and B55 segments. The extremum relative displacement is −8.4 mm between the flat and inclined segments at the exit.

Generally, the largest relative displacements take place at case I, while those at case III are larger than those at case II. The complex soil foundation obviously affects the seismic displacement, and the relative displacement reaches the peak between segments B43 and B44, B47 and B48, B54 and B55 on the mutation region of soil layers. The large relative displacement also appears between inclined and flat segments at entrance and exit ends. The sealed-joints of these locations will be easily broken under seismic action.

## Conclusions

A comprehensive FE model of shallow-buried large-scale prestressed concrete inverted-siphon is built in this paper combined with the theories of boundary interaction, fluid–structure interaction and joints interaction between adjacent segments, and the seismic behaviors of the inverted-siphon under multiple actions are analyzed. Conclusions can be drawn as follow:

The vibration frequency and mode of inverted-siphon are obviously influenced by the thickness of overburden soil compared with the water flow. The vibration frequency increases with a larger vibration amplitude as the decrease of the thickness of overburden soil, while it increases when the water flows through one box instead of three boxes. The former ranks of vibration mode are mainly vertical vibration with obviously relative deformation between adjacent segments.

Suffered from the transversal and vertical seismic excitations, the walls of inverted-siphon are mainly in compression at lower stress level. The structure stresses are obviously influenced by the thickness of overburden soil compared with the water flow. Large transversal tensile stresses appear on the top-plate bottom-surface in side boxes and the bottom-plate upper-surface in three boxes, which reach 3.6 MPa and 3.1 MPa at cases II and III, respectively. This leads a possible of concrete cracking due to the earthquke.

The relative displacement between adjacent segments increases with the decreased thickness of overburden soil and the increase of water flow. It reaches the extremum when the adjacent segments locate on a complex geology. The joints of flat and inclined segments also present a large deformation. In these joints, the risk of water leakage increases due to the tearing broken of sealed-joints made of rubber belt.

The seismic behaviors of shallow-buried large-scale prestressed concrete inverted-siphon change with the structure-soil and structure-water interactions, and affected by the thickness of overburden soil and the water flow in service period. The study is necessary to reveal the weakness of seismic resistance and take specific measures for preventing the earthquake damage.

## Data Availability

The datasets used and/or analysed during the current study available from the corresponding author on reasonable request.

## References

[1] Zhao S, Li X, Yan Z, Liu X (2008). Research and Engineering Application of High-performance Ring Unbonded Prestressed Concrete Technology.

[2] Li X, Chen Z, Zhao Y, Zhao S (2019). Unbonded Prestressed Concrete Lining of Pressure Tunnel.

[3] Zhao S, Qiao Y, Ma S (2011). Prestressed Concrete Technology for Inverted-siphon Structure.

[4] Yao G, Chen Z, Yan Z, Li X (2020). Analysis of loading behavior of prestressed concrete lining structure for water convey tunnel under high-water pressure. Yangtze River.

[5] Zhao S, Li X, Hu Z (2006). Experimental study on simulated model of large prestressed concrete inverted siphon. J. Hydroelectr. Eng..

[6] Pei S, Liu S, Zhao S, Li X (2006). Influences of foundation changes on behaviors of large prestressed concrete inverted siphon. J. Yangtze River Sci. Res. Inst..

[7] Kaur K, Laanearu J, Annus I (2017). Numerical study of Tallinn storm-water system flooding conditions using CFD simulations of multi-phase flow in a large-scale inverted siphon. IOP Conf. Series: Mater. Sci. Eng..

[8] Jin S, Liu H, Ding W, Shang H, Wang G (2018). Sensitivity analysis for the inverted siphon in a long distance water transfer project: An integrated system modeling perspective. Water.

[9] Guo L, Wang L, Xu Q (2010). Simulation and application on concrete temperature control measures for inverted siphons in winter. Transact. CSAE.

[10] Fu H, Yang K, Guo X, Guo Y, Wang T (2015). Safe operation of inverted siphon during ice period. J. Hydrodyn..

[11] Fu H, Yang K, Guo Y, Wang T, Guo X (2013). An experimental study on ice jam prevention of typical inverted siphon for South-to-North water diversion project. Adv. Water Sci..

[12] National Energy Administration (2015). Code for Seismic Design of Hydraulic Structures of Hydropower Proiect (NB 35047–2015).

[13] Li Z, Chen Y, Shi Y (2017). Numerical failure analysis of a continuous reinforced concrete bridge under strong earthquakes using multi-scale models. Earthq. Eng. Eng. Vib..

[14] Emadoddin MF, Shahrokh M (2017). An investigation of the seismic behavior of a deck-type reinforced concrete arch bridge. Earthq. Eng. Eng. Vib..

[15] Lysmer J, Kulemeyer RL (1969). Finite dynamic model for infinite media. J. Eng. Mecha. ASCE.

[16] Joyner WB, Chen ATF (1975). Calculation of nonlinear ground response in earthquake. Bull. Seismol. Soci. Am..

[17] Deeks AJ, Randolph MF (1994). Axisymmetric time-domain transmitting boundaries. J. Eng. Mecha..

[18] Liao Z (2002). Introductory Theory of Wave Motion for Engineering, 2nd edit.

[19] Liu J, Wang Z, Du X, Du Y (2005). Three-dimensional visco-elastic artificial boundaries in time domain for wave motion problems. Eng. Mecha..

[20] Du X, Zhao M (2006). Analysis method for seismic response of arch dams in time domain based on viscous-spring artificial boundary condition. J. Hydraul. Eng..

[21] Ma S, Cao L, Xiao M, Zhang Z (2012). Method for setting artificial boundary used in time history analysis of underground caverns at great depth. J. Sichuan Univ. (Adv Eng. Sci.).

[22] He C, Zhou S, Di H, Guo P, Xiao J (2018). Analytical method for calculation of ground vibration from a tunnel embedded in a multi-layered half-space. Comput. Geotech..

[23] He C, Zhou S, Guo P, Di H, Zhang X (2018). Analytical model for vibration prediction of two parallel tunnels in a full-space. J. Sound Vib..

[24] Westergaard HM (1933). Water pressure on dams during earthquakes. Trans. ASCE.

[25] Buldgen L, Caprace JD, Rigo P, Sourrne HL (2017). Investigation of the added mass method for seismic design of lock gates. Eng. Struct..

[26] Chorpa AK, Chakrabarti P (1981). Earthquake analysis of concrete gravity dams including dam-water-foundation soil interaction. Earthq. Eng. Struct. Dynam..

[27] Li Y, Lou M, Shang W, Zhu J (2003). Displacement-based fluid finite element for seismic-resistant analysis of large-scale aqueduct. J. Hydraul. Eng..

[28] Gong Y, Su H, Cui J (2011). Fluid-solid coupling analysis of dam-reservoir interaction. J. Yangtze River Sci. Res. Institute.

[29] Elansary AA, Damatty AAE (2018). Seismic analysis of liquid storage composite conical tanks. Eng. Struct..

[30] Hariri-Ardebili MA, Seyed-Kolbadi SM (2015). Seismic cracking and instability of concrete dams: Smeared crack approach. Eng. Fail. Anal..

[31] Hariri-Ardebili MA, Kianoush MR (2014). Integrative seismic safety evaluation of a high concrete arch dam. Soil Dyn. Earthq. Eng..

[32] Zhang Q, Li D, Wang F, Li B (2017). Numerical simulation of nonlinear structural responses of an arch dam to an underwater explosion. Eng. Fail. Anal..

[33] Chen Z, Zhao S (2012). Dynamic finite element analysis of solid and fluid-saturated porous solid coupling. Yellow River.

[34] Zhao S, Liu Z, Liu S (2007). Study on structural dynamic behavior of roof truss-arched aqueduct. Water Resour. Hydropower Eng..

[35] Ding H, Tong L, Xu C, Zhao X, Nie Q (2019). Dynamic responses of shallow buried composite cylindrical lining embedded in saturated soil under incident P wave based on nonlocal-Biot theory. Soil Dyn. Earthq. Eng..

[36] SL 191–2008, *Design code for hydraulic concrete structures*, (Beijing: China Waterpower Press, China, 2009).

[37] Oudni N, Boua Y (2015). Response of concrete gravity dam by damage model under seismic excitation. Eng. Fail. Anal..

[38] Abate G, Massimino MR (2017). Numerical modelling of the seismic response of a tunnel-soil-aboveground building system in Catania. Bull. Earthq. Eng..

[39] Li P, Song E (2015). Three-dimensional numerical analysis for the longitudinal seismic response of tunnels under an asynchronous wave input. Comput. Geotech..

[40] Xu L, Ye Z, Ren Q, Zhang L (2013). FEM analysis of dynamic response of buried fiber reinforced plastic matrix pipe under seismic load. Math. Probl. Eng..

[41] Huang J, Du X, Zhao M, Zhao X (2017). Impact of incident angles of earthquake shear (S) waves on 3-D non-linear seismic responses of long lined tunnels. Eng. Geol..

